# Compensation of adverse growing media effects on plant growth and morphology by supplemental LED lighting

**DOI:** 10.1371/journal.pone.0291601

**Published:** 2023-09-14

**Authors:** Jan Andreas Solbach, Andreas Fricke, Hartmut Stützel

**Affiliations:** Vegetable Systems Modelling Section, Institute of Horticultural Production Systems, University of Hannover, Hannover, Germany; Wageningen University, NETHERLANDS

## Abstract

There is an increasing interest in alternatives to peat in growing media due to environmental constraints. However, plants grown in peat substitutes often show impaired growth compared to plants grown in peat-based media. Hence, it would be interesting to know whether these deficiencies can be compensated by supplementing other growth factors, e.g. light. The present study aims to investigate the interactive nature between growing media and supplemental lighting on plant growth and morphology, and to examine whether supplemental light emitting diode (LED) lighting may compensate adverse growing media effects. Basil (*Ocimum basilicum* L.) and Chinese cabbage (*Brassica rapa* subsp. *pekinensis*) were grown in different growing media consisting of peat, green compost, coconut pulp, wood fibre, perlite and sphagnum moss under blue, red and far-red supplemental LED lighting. We found significant interactions between growing media and supplemental photosynthetically active radiation (PAR) on plant growth, morphology and development. At low light intensities, peat-based and substituted growing media performed similarly, whereas with increasing light intensities the peat-based growing media significantly outperformed their alternatives. The substrate choice determines the required amount of supplemental light to compensate for adverse growing media effects and the amount varies depending on plant species and season. Thereby, it was indicated that red light alleviates adverse growing media effects best. We also found that far-red light is not effective when background PAR is low and becomes more effective under high background PAR. The implications and prospects of the results are discussed.

## 1 Introduction

Peat is the main component used in horticultural growing media in Europe [1]. However, due to the negative impact of unsustainable peat extraction including the destruction of peat bogs as carbon sinks and damaged ecosystems [2], increased political pressure to achieve EU sustainability objectives [3] and rising awareness among consumers for alternative growing media [4] the horticultural growing media industry is forced to search for alternative materials. Promising peat substitutes include composts from green waste, coconut coir, wood fibre, sphagnum moss, perlite and biochar, among other materials (see [1, 5] for comprehensive review on peat substitutes). However, growing plants solely in peat substitutes or substituting major proportions of peat with alternative materials may lead to impaired growth depending on the plant species and substitutes used [6–12]. Hence, further research on alternative constituents and solutions are required to enable the substitution of larger proportions of peat in horticultural growing media.

An alternative solution may be to include the use of supplemental light emitting diode (LED) lighting. Narrow-spectrum lighting tailored to crops allows the manipulation of plant growth processes like leaf expansion or photosynthesis [13]. Red light (600–700 nm) is considered the most efficient in plant photosynthesis [14–16]. However, growing plants solely under red light reduces growth and photosynthesis [17, 18], also known as the red-light syndrome [19–21]. Adding blue light (400–500 nm) may suppress these symptoms [19–21]. Blue light is essential for proper functioning of photosynthesis [19] and involved in various physiological key functions including light-induced chloroplast movement and stomatal opening [22, 23]. Far-red light (700–800 nm) is generally considered inefficient for plant photosynthesis. However, latest findings demonstrated that far-red photons interact synergistically with shorter wavelengths and may drive photosynthesis equally efficient [24, 25]. Far-red light is also involved in phytochrome mediated phenotypic adaptation known as the ’shade avoidance syndrome’ [26]. Shade avoidance reactions may occur when plants perceive a low red to far-red light ratio (R:FR). Responses include rapid elongation of stems and leaves, increased specific leaf area (SLA) and a reduction in leaf chlorophyll content [27].

Recent research on crop responses to supplemental LED lighting under controlled environments mainly focused on optimization of light spectra and photon flux density (e.g. [19, 28–36]). However, fewer studies investigated the interactions of supplemental lighting with other environmental factors such as air temperature, fertilizer concentration and growing season under controlled environments [37–40].

Here, we studied the compensatory potential of supplemental LED lighting on plant growth in different growing media under greenhouse conditions. Our aim was to understand the response of plant growth, particularly photosynthesis and leaf expansion, to supplemental lighting in plants growing in different media and seasons in order to evaluate whether supplemental LED lighting may compensate for possible adverse growing media effects. Chinese cabbage and basil were used as test crops.

## 2 Material and methods

Two experiments were conducted to study compensation by supplemental light dose and quality (Experiment I) and during different seasons (Experiment II).

### 2.1 Experiment I (EXP 1)

EXP 1 took place from 15^th^ January to 13^th^ February 2019 in a greenhouse at the experimental site of Leibniz University Hannover, Hannover, Germany. Chinese cabbage (*Brassica rapa* subsp. *pekinensis* ‘Kilakin’, Syngenta Agro GmbH, Maintal, Germany) was sown in 10-cell trays (5 cm H × 4.5 cm W × 4.5 cm L per cell) containing two different growing media: Potgrond H (Potgrond H90, Klasmann-Deilmann GmbH, Geeste, Germany; control) and 20% green compost mixed with 80% wood fibre (TerrActiv^®^ and GreenFibre^®^, both Klasmann-Deilmann GmbH, Geeste, Germany; substitute). Prior to the start of the trial, fertilizer was added to the substitute to match nutrient contents of Potgrond (S1 Table). To offset possible N-immobilization in the growing media, we used N-impregnated wood fibre and additionally fertigated by using a 6.86 g L^-1^ ammonium nitrate solution throughout the growing cycle. Trays containing both substrate mixtures were arranged within five supplemental LED light intensity levels (183, 111, 44, 15 and 5 μmol m^-2^ s^-1^), and within three light quality treatments (S1 Fig), namely blue (440 nm + 465 nm), red (660 nm) and white plus far-red (730 nm, Table 1). The white plus far-red light treatment included white light to ensure the same amount of PAR in all light treatments and is referred to as ‘far-red’ light treatment in the following. Light spectral curves are shown in S2 Fig. Light quality treatments were arranged in a randomized complete block design with four blocks (replications). Therefore, four benches (4.8 m × 2 m) were each evenly divided into three compartments (1.6 m × 2 m) by black plastic sheets (2 m × 0.40 m, Lux Baufolie, Emil Lux GmbH & Co. KG, Wermelskirchen, Germany) to avoid light quality mixing. Light was provided by LEDs (LED-KE 300, DH Licht GmbH, Wülfrath, Germany) that were switched on at 5 am and off at 10 pm Greenwich Mean Time. PAR and spectral data was recorded with a spectrometer (USB4000, OceanOptics, Inc., Florida, USA). Greenhouse day and night heating setpoints were set to 20°C and 16°C, respectively. The ventilation setpoint was 24°C. Average natural daily light integral was 2.98 mol m^-2^ s^-1^ during plant cultivation within the greenhouse. Furthermore, two water supply treatments were imposed by withholding water till thresholds of 70% (control) and 50% (stressed) substrate specific water holding capacity (WHC, see section 2.3) were reached. Trays including plants were weighted daily to check whether the thresholds were reached. When the respective WHC was reached, plants were fertigated up to 100% WHC. These fertigation cycles started 17 days after sowing and continued until the end of the experiment.

**Table 1:** Supplemental light distribution within the white plus far-red light treatments.

PAR^w^	Spectral composition				Spectral ratio	
μmol m^-2^ s^-1^	400–500 nm	500–600 nm	600–700 nm	700–800 nm^x^	FR/PAR^y^	R/FR^z^
**183**	32.2%	42.5%	25.3%	49.0%	1: 2.0	0.11
**111**	28.2%	44.5%	27.2%	55.6%	1: 1.8	0.11
**44**	24.2%	46.8%	29.0%	63.6%	1: 1.6	0.09
**15**	21.3%	48.7%	30.0%	66.8%	1: 1.5	0.10
**5**	20.3%	49.7%	30.0%	65.8%	1: 1.5	0.09

^w^ Mean values of six PAR (400–700 nm) measurements within each treatment level

^x^ Proportion of far-red (700–800 nm) to total PAR (400–700 nm)

^y^ Ratio of far-red (700–800 nm) to PAR (400–700 nm)

^z^ Ratio of red to far-red calculated according to [26]

### 2.2 Experiment II (EXP 2)

In experiment 2, four trials were conducted in a greenhouse at the experimental site of Leibniz University Hannover, Institute of Horticultural Production Systems, Vegetable Systems Modelling Section, Hannover, Germany. Seeds of Chinese cabbage (*Brassica rapa* subsp. *pekinensis* ‘Kilakin’, Syngenta Agro GmbH, Maintal, Germany) and basil (*Ocimum basilicum* L. cv. ‘Edwina’, Enza Zaden Beheer B.V., Enkhuizen, Netherlands) were sown in 10-cell trays (5 cm H × 4.5 cm W × 4.5 cm L per cell) containing three different growing media:

- 100% white peat (control)
- 30% coir pith, 20% wood fibre, 50% perlite (Growing medium I)
- 15% green compost, 35% wood fibre, 20% perlite, 30% sphagnum moss (Growing medium II).

All raw materials were scoured from Klasmann-Deilmann GmbH, Geeste, Germany and self-mixed. Prior to the start of each trial, all growing media were fertilized up to approximate the nutrient level of the control growing medium (S1 Table). The heating setpoints of the greenhouse was set to 20°C day/16°C night temperature and vents opened at 24°C. Trials with Chinese cabbage took place from 25^th^ April to 16^th^ May (Spring) and 19^th^ June to 10^th^ July (Summer), and those with basil from 31^st^ July to 4^th^ September (Summer) and 25^th^ September to 30^th^ October 2017 (Autumn, Table 2). Growing media were watered according to their substrate specific WHC. Trays were weighted daily and fertigated whenever the media water content dropped below the threshold of 70% of their WHC with a nutrient solution containing 2 g L^−1^ Ferty^®^ 2 Mega fertilizer (Planta Düngemittel GmbH, Regenstauf, Germany). The light treatments were applied with LED lamps equipped with blue, red and far-red LEDs (LED-KE 300, DH Licht GmbH, Wülfrath, Germany) that were placed in 1.6 m distance next to each other and 1 m above each bench. Peak wavelengths of blue, red and far-red LEDs were 440 nm + 465 nm, 660 nm and 730 nm, respectively. LEDs were not separated by any means and thereby created gradients of light intensities and qualities which allowed the study of light dose-responses of varying light qualities (S3 Fig). LEDs were turned on at 5 am and off at 11 pm Greenwich Mean Time (18 h of supplemental light). Light spectral data was captured with a spectrometer (USB4000, OceanOptics, Inc., Florida, USA). Trays were arranged along the light gradient in 19 positions (S3 Fig), each including the three growing media in a randomized fashion. The trial trays were surrounded by an additional row of trays to avoid border effects.

**Table 2:** Summary of the growing conditions present during the four greenhouse trials.

Season	Chinese cabbage		Basil	
	Spring	Summer	Summer	Autumn
**Air temperature [°C]**	21.1	23.5	22	17.8
**Natural DLI**^x^ **[mol PAR m**^**-2**^ **d**^**-1**^**]**	13.7	16.6	13.1	5.4
**SL+NL**_**max**_^y^ **[mol PAR m**^**-2**^ **d**^**-1**^**]**	31.7	43.2	30.0	20.9
**SL/NL** ^z^	0.73	0.6	0.76	1.85

^x^ Average daily light integral inside the greenhouse

^y^ Maximum supplemental plus natural daily light integral directly under the LEDs

^z^ Maximum supplemental plus natural light directly under the LEDs

### 2.3 Determination of growing media water holding capacity

Water holding capacity (WHC) was determined for each growing medium using the following procedure: Six trays were filled with growing media and then waterlogged. Afterwards, the trays were covered with black plastic film to minimize evaporation and allowed to drain for 24 hours. Thereafter, single trays were weighted to determine their weight at full WHC. Trays were then dried in an oven for 96 hours to determine the dry weight of the growing medium. WHC was calculated as the difference between the medium weight at full water capacity and the dry weight of medium divided by the medium weight at full water capacity. The mean value of the six trays were used to calculate final WHC.

### 2.4 Collection of growth and morphological data

Measurements were taken from 6 and 10 biologically distinct plants per tray in EXP 1 and EXP 2, respectively. Plants were cut above the growing media surface and plant fresh weights were taken first. The number of leaves (> 1 cm) were counted. Leaf areas were determined subsequently using a leaf area meter (LI-3100C Area Meter, LI-COR, Lincoln, NE, USA). Afterwards, the samples were dried in an oven at 70°C for at least 72 hours to determine dry weights (TU-2, Heraeus Holding GmbH, Hanau, Germany). Specific leaf area (SLA) was calculated by dividing leaf area by leaf dry weight. In addition, hypocotyl length of Chinese cabbage was collected with a ruler. Pricked out plants were not considered for data sampling.

### 2.5 Estimation of compensation requirements of adverse growing media effects

First, ten plants were averaged to give one dry weight value per growing media and PAR level (S2 Table, Experiment II). There were 19 and 17 PAR levels (ranging from 3 to 142 μmol m^-2^ s^-1^) in total for Chinese Cabbage and basil, respectively (S4A Fig). These data points were used to estimate missing dry weights per μmol PAR m^-2^ s^-1^ for each growing media and season by interpolating data assuming a linear relationship between dry weight and μmol PAR m^-2^ s^-1^ (S4B Fig). The PAR intensity needed to compensate was then obtained by estimating the PAR intensity needed to reach the same dry weight as the control (S4C Fig, i.e. where the red horizontal line intersects with the interpolated line). In case of basil during summer (Fig 2B) data was not only interpolated but extrapolated up to 500 μmol PAR m^-2^ s^-1^, assuming a linear relationship between dry weight and PAR intensity as well to make the trend clearer. For Chinese Cabbage during summer (Fig 3B) only two data points are shown as the amount of PAR needed to compensate would exceed 500 μmol m^-2^ s^-1^ by a large margin. Compensation by light quality was estimated in the same way but based on 5 PAR levels instead.

### 2.6 Statistics

All statistical tests were performed in R [41]. Data from multiple biological replicates per treatment was first averaged to give one response per treatment and per replication. Means and standard errors of means (SEM) were calculated next based on four repetitions per treatment (n = 4, S2 Table). Afterwards the *lmer* function of the lme4 package [42] was used to fit a linear mixed model to the data. Analysis of variance (ANOVA) was performed to examine differences among treatment effects and their interactions at the 5% probability level. Results of ANOVA are shown in S3 Table. The emmeans package was then used to estimate marginal means using the *emmeans* function [43]. Data was checked for normal distribution and homogeneity prior to analysis.

## 3 Results

### 3.1 Interaction between supplemental light dose and growing media

**Fig 1:**
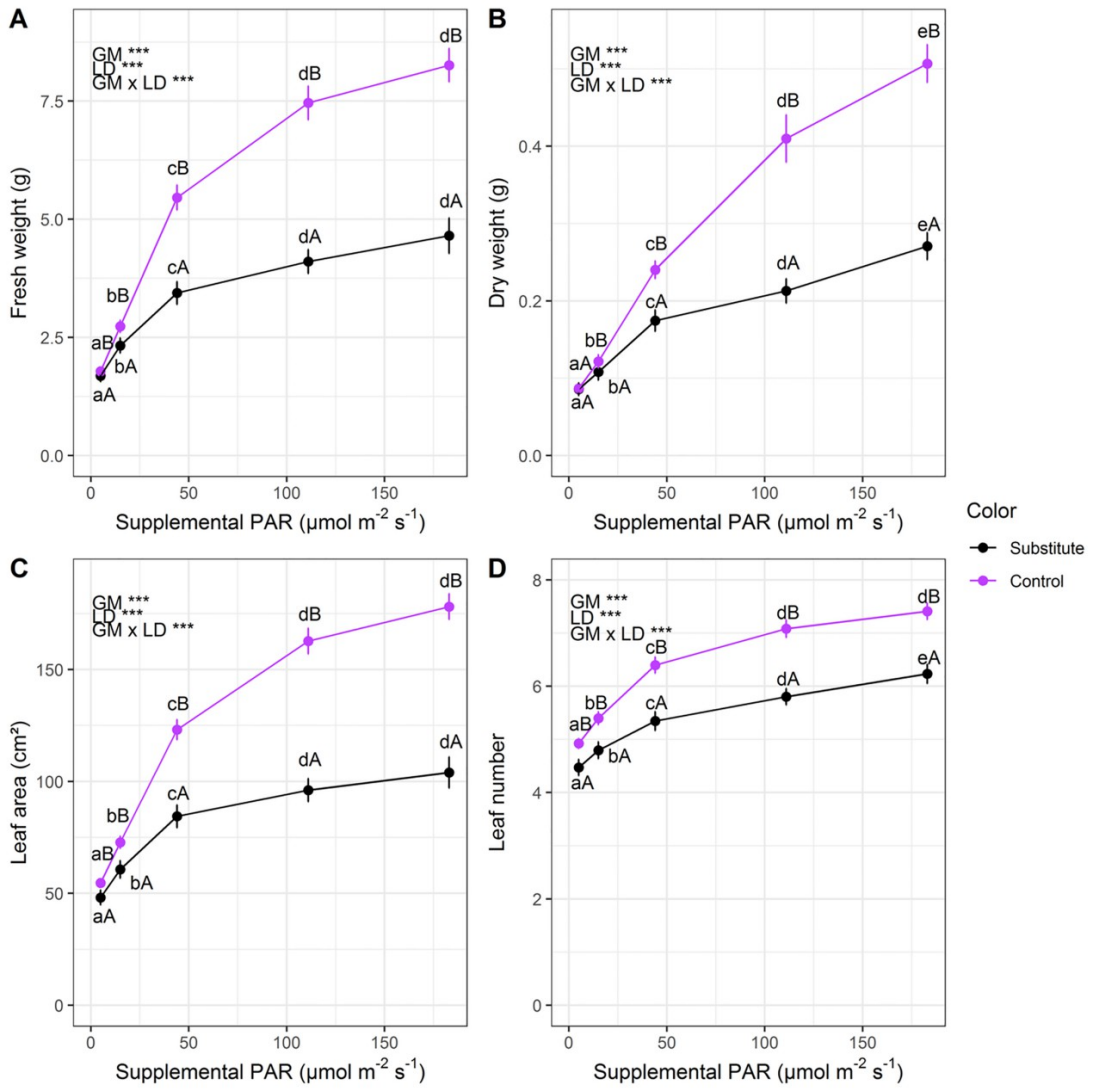
Effects of supplemental light dose and growing media on fresh weight (A), dry weight (B), leaf area (C) and leaf number (D) of Chinese cabbage grown in two media (Substitute: 20% compost and 80% wood fibre; Control: Potgrond H) under LEDs in a greenhouse. Levels of significance for growing media (GM), light dose (LD) and their interaction (GM x LD) are depicted (***, P<0.001). Different lower-case and upper-case letters indicate significant differences among light doses within growing media treatments and among growing media within light dose treatments, respectively. Error bars indicate SEM. Lines connecting observations are for visualization only.

All investigated plant traits were significantly affected by light dose and substrate choice and showed significant interactions (Fig 1). An increase in supplemental light dose linearly increased plant fresh weight, dry weight, leaf area and leaf number in both substrates. Depending on the growing media, increasing supplemental PAR from 5 to 183 μmol m^-2^ s^-1^ resulted in a 2.5 to 4-fold increase in plant fresh weight, 4 to 5-fold increase in dry weight, 2 to 3-fold increase in leaf area and further accelerated leaf development (Fig 1). Growth of plants in peat substitutes was significantly impaired compared to plants grown in a peat growing medium. Interestingly, at lower light intensities plants grown in both growing media performed rather similar, whereas with increasing light dose the gap between growing media increased, indicating the interactive effect of growing medium and supplemental light dose on plant growth, morphology, and development.

### 3.2 Compensatory effects of supplemental light dose

In most cases, compensation of adverse effects of peat substituted growing media was possible with additional supplemental light. The required amount of additional light thereby depended on the growing media, season, and plant species.

**Fig 2:**
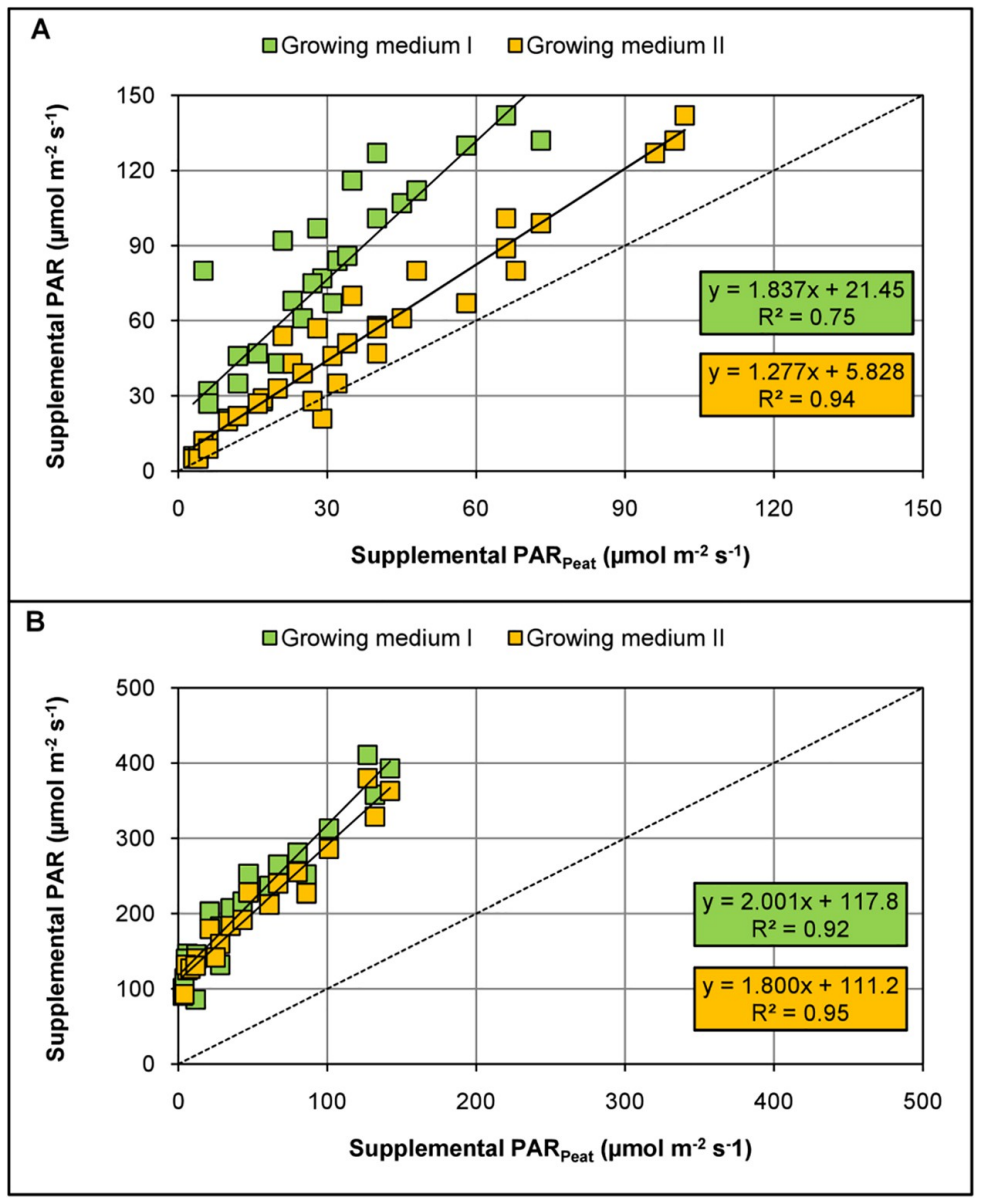
Comparison of two peat substituted growing media as compared to a peat-based growing media (control) on dry weight of basil grown under supplemental LED lighting during autumn (A) and summer (B). Values indicate the amount of supplemental PAR required to reach the same dry weight as the control. The green squares indicate growing medium I (composed of 30% coconut pulp, 20% wood fibre, 50% perlite) and the orange squares indicate growing medium II (composed of 15% green compost, 35% wood fibre, 20% perlite, 30% sphagnum moss). The solid lines show the linear regressions *y* = *ax* + *b*. Dotted grey lines represent 1:1 lines.

Basil performed better in growing medium II than in medium I as indicated by the lower slopes of the linear regression lines (Fig 2). However, plants produced less dry weight in both substitutes leading to steeper regression slopes (i.e., above 1) compared to a peat-based growing media. As an example, for basil during autumn, these steeper slopes mean that for 1 μmol PAR m^-2^ s^-1^ applied to the control (peat), 1.8 μmol PAR m^-2^ s^-1^ and 1.3 μmol PAR m^-2^ s^-1^ need to be given to growing medium I and II, respectively, to get the same dry weight increase. Beforehand, 5.8 μmol PAR m^-2^ s^-1^ and 21.45 μmol PAR m^-2^ s^-1^ are necessary to bring the plants in growing medium I and II, respectively, to the same weight reached by the control plants without supplemental light. Furthermore, compensation requires more supplemental light during high light conditions (summer) compared to low light conditions (autumn) as indicated by the steeper slopes and higher intercepts of the regressions in summer. The amount required for compensation is 8.2% to 29.1% lower during autumn than in summer depending on the growing media. These observations concur with the previously described interactive effect of supplemental light dose and growing medium (see section 3.1).

**Fig 3:**
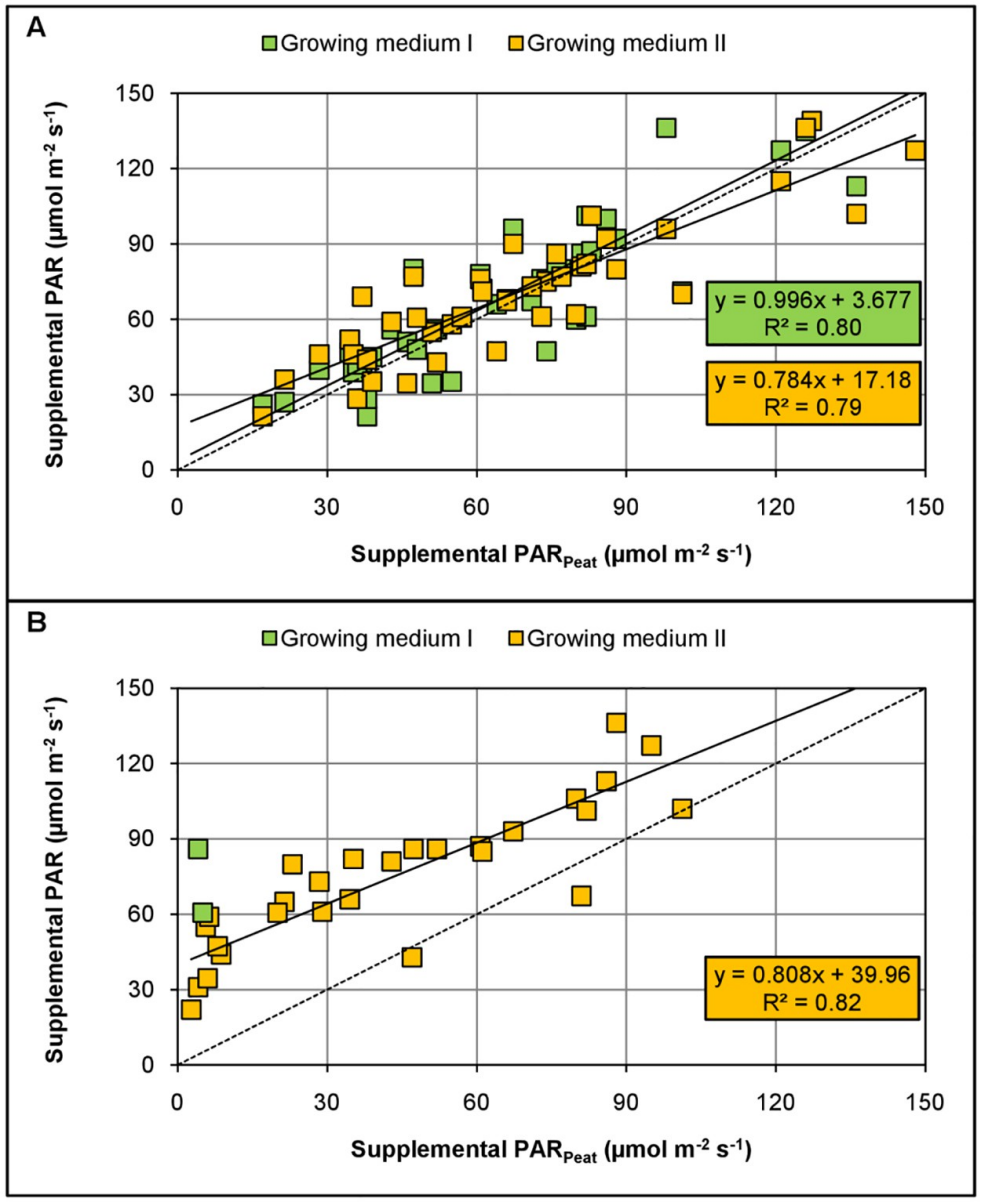
Comparison of two peat substituted growing media as compared to a peat-based growing media (control) on dry weight of Chinese cabbage grown under supplemental LED lighting during spring (A) and summer (B). Values indicate the amount of supplemental PAR required to reach the same dry weight as the control. The green squares indicate growing medium I (composed of 30% coconut pulp, 20% wood fibre, 50% perlite) and the orange squares indicate growing medium II (composed of 15% green compost, 35% wood fibre, 20% perlite, 30% sphagnum moss). The solid lines show the linear regressions *y* = *ax* + *b*. Dotted grey lines represent1:1 lines.

On the other hand, Chinese cabbage produced similar biomasses in both peat substitutes as compared to the peat-based growing medium during low light conditions (Fig 3). Regression slopes are rather similar and closely follow the 1:1 line. Hence, there is no need for compensation during spring. During summer, however, growing medium II strongly outperformed growing medium I. These findings demonstrate a seasonal dependency of the different substrates and indicate a plant species-specific behavior.

### 3.3 Interactive effects of far-red light and PAR

**Fig 4:**
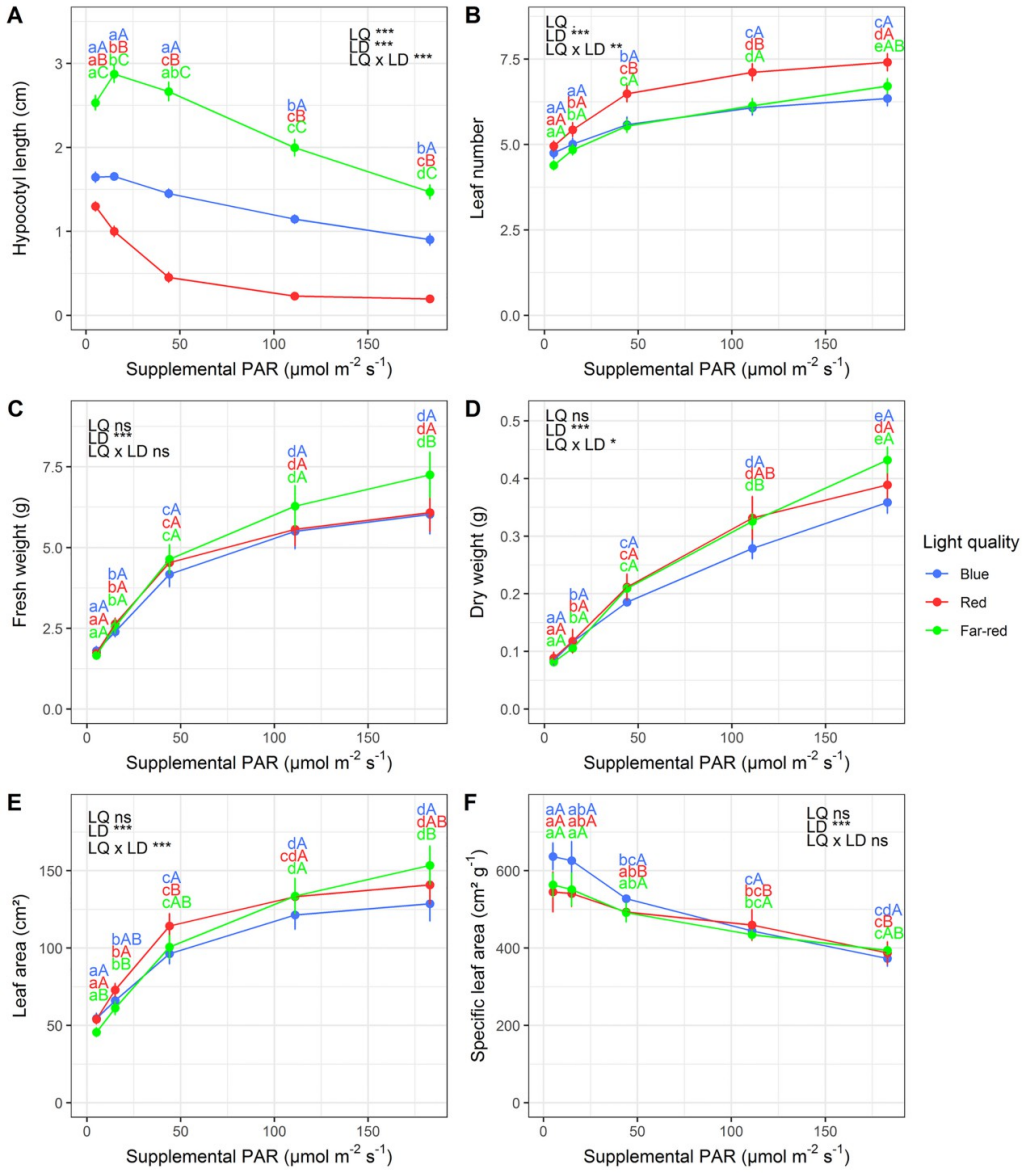
Effects of supplemental light dose and light color on hypocotyl length (A), leaf number (B), fresh weight (C), dry weight (D), leaf area (E) and specific leaf area (F) of Chinese cabbage grown under LEDs in a greenhouse. Different lower-case and upper-case letters indicate significant differences among light doses within light quality treatments and light qualities within light dose treatments, respectively. Data was averaged over growing media. Levels of significance for light quality (LQ), light dose (LD) and their interaction (LQ x LD) are depicted (***, P<0.001; **, P<0.01; *, P<0.05; P<0.1; ns, not significant). Error bars indicate SEM. Lines connecting observations are for visualization only.

For Chinese cabbage all investigated plant traits were significantly affected by light dose. An increase in light dose was associated with an increase in leaf number, fresh weight, dry weight, leaf area, light interception as well as a decrease in hypocotyl length, SLA, LUE, and leaf area per incident light (Fig 4 and S5 Fig). There was no effect of light quality on plant growth, morphology, and development, except for hypocotyl length. However, although there was almost no direct effect of light quality on these traits, there were significant interactions between light quality and light dose (Fig 4 and S5B and S5C Fig).

Supplement of far-red light led to more elongated plants compared to blue and red light (Fig 4A), but this effect depended on the applied light dose. Red light induced a sharp decline in hypocotyl length with increasing light dose. There was a similar tendency in plants grown under blue light, but the effect was significantly lower. On the other hand, hypocotyl length of plants grown under far-red light peaked under low light conditions and then further decreased with increasing light dose. Besides its effect on hypocotyl length, light quality tended to affect leaf development as well (p<0.1, Fig 4B). Leaf number tended to be generally higher under red light compared to blue and far-red light. Although leaf number tended to be greater under red light it was not reflected in increased biomass production (Fig 4C and 4D) compared to the other light quality treatments.

Interactions between light quality and light dose could be seen in leaf number, dry weight and leaf area (Fig 4B, 4D and 4E). Under low light doses, plants grown under far-red light generally performed worse than or similar to plants grown under red and blue light. With increasing supplemental light, however, plants grown under far-red light started to outperform those under the other light treatments. Under high supplemental light dose they even exceeded plants grown under blue light (Fig 4D and 4E). This indicates a dependency of far-red light effects on total light. The higher efficiency of far-red light was not associated with a change in leaf thickness (Fig 4F) nor LUE (S5A Fig) but with an altered light interception (S5B Fig) due to increased leaf area per incident light (S5C Fig) under increasing light dose.

### 3.4 Compensatory effects of supplemental light quality

**Fig 5:**
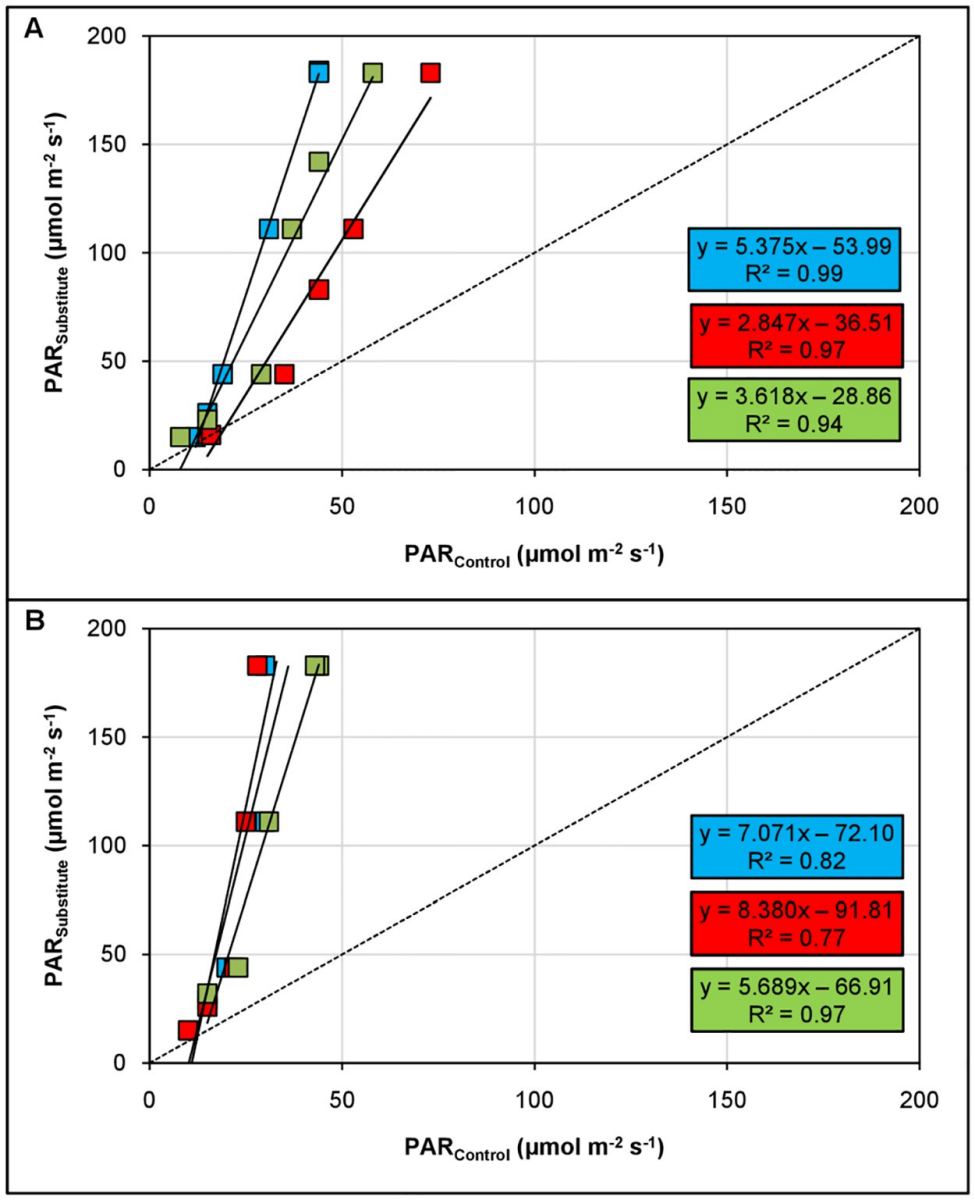
Effect of blue, red and white plus far-red light on dry weight (A) and leaf area (B) of Chinese cabbage grown in a peat substituted growing media (Substitute: 20% compost and 80% wood fibre) as compared to a peat-based growing media (Control: Potgrond H). Values indicate the amount of supplemental PAR of a specific wavelength required to reach the same dry weight and leaf area as the control. The solid lines show the linear regressions *y* = *ax* + *b*. Dotted grey lines represent 1:1 lines.

Different light qualities compensate for adverse growing media effects to various degrees (Fig 5). Red light alleviates adverse growing medium effects on dry matter production best, followed by white plus far-red light and blue light as indicated by the differences in slopes of the linear regression lines (Fig 5A). Regarding leaf area, far-red light was slightly more efficient in compensation compared to red and blue light (Fig 5B). The higher efficiency (i.e., lower slope of the linear regression line) of red light to compensate adverse growing media effects on dry weight was associated with increased plant dry weights, altered SLA (thicker leaves) and increased light use efficiency under red light compared to blue and far-red light (S6 Fig). As such, the amount of supplemental PAR of a specific wavelength required to reach the same dry weight as compared to the peat-based growing medium is about 2-fold, 3-fold and 4-fold higher under red light, white plus far-red light and blue light, respectively.

## 4 Discussion

### 4.1 Better external growing conditions favor peat-based growing media

Chinese cabbage grown in the peat-free growing medium exhibited significantly impaired growth and development compared to its peat-based counterpart (Fig 1). Reduced plant growth in peat-reduced or peat-free growing media often occurs due to suboptimal plant nutrition, chlorophyll content, and consequently photosynthesis [11, 44, 45]. Growing media composition can also adversely affect germination, seedling growth and morphology [46–51] that are prerequisites for further vigor plant growth. The substitute growing media consisted of 20% green compost and 80% wood fibre (EXP I). Wood fibres are generally characterized by high air holding capacity, low water holding capacity and good re-wettability. Therefore, they are often mixed with other components [1, 52] and seldomly used as a stand-alone growing medium [5]. However, they may also contain phytotoxins that negatively influence germination and early plant growth and development as observed in pine tree fibre growing media [53]. The second component in the mixture, green compost, is generally characterized by biological instability, phytotoxicity, very high electrical conductivity, high pH, K_2_O and organic matter content [1, 5, 52]. Increased pH values and K_2_O contents could also be found in our growing media containing green compost (S1 Table). Additionally, the use of green compost may lead to different micronutrient uptakes such as copper, zinc, nickel, and manganese due to nutrient imbalance which involves magnesium, potassium, and phosphorus. Consequently, the export of photosynthates and the allocation of nutrients may be disturbed [7]. The highly microbial active green compost coupled with the high proportion (80%) of wood fibre in the growing medium may have aggravated effects and might therefore explain the observed reduced growth and development.

Interestingly, under low light conditions peat-based and substituted growing media performed rather similar, whereas with an increase in light dose the peat-based growing media strikingly outperformed its alternative. The observed interactions between growing medium and light dose might therefore be an indirect effect of reduced growth and photosynthesis during seedling establishment in peat-free growing media. Plants grown in the peat-free media already started to lag behind during early growth. As plants were watered accounting for growing media specific WHC’s and we did not observe any wilting, drought stress during early seedling establishment may be discarded as an explanation. Moreover, since the gap widened with increasing light dose (Fig 1) it is indicated that the plants could not equally well utilize the more favorable growth environment compared to the peat-based growing medium.

These findings highlight the importance of the growing medium when supplemental LED lighting is applied and suggest, in a nutshell, that better external growth conditions favor peat-based growing media compared to peat-free growing media.

### 4.2 Supplemental lighting as a compensation tool against adverse growing media effects

We showed that the addition of supplemental lightning may compensate adverse growing media effects. Compensation thereby varies between seasons as more supplemental light is required during summer compared to autumn and spring (Figs 2 and 3). It was recently demonstrated that supplemental LED lighting efficiencies during greenhouse production are directly affected by the growing season. Supplemental lighting efficiencies are thereby lower during high light conditions compared to low light conditions [40].

Thus, it is logical that the required amount of supplemental light to compensate for adverse growing media effects is higher under abundant natural light than light limited conditions due to altered efficiencies of the supplemental light as observed in this study. Moreover, the required amount of supplemental light to compensate for adverse growing media effects was approximately two-fold higher in basil (Fig 2) compared to Chinese cabbage (Fig 3). Hence, the amount of additionally required light strongly varies depending on the cultivated plant species making the plant species a crucial factor influencing compensation.

Furthermore, our results revealed that the use of a specific wavelength (i.e., red light on dry weight and far-red light on leaf expansion) may compensate adverse growing media effects more efficiently (Fig 5). The slightly higher efficiency of far-red light to compensate adverse growing media effects on leaf area was associated with increasing light interception compared to the other light quality treatments (S5B Fig). Addition and substitution of far-red light by PAR is generally known to enhance leaf expansion resulting in increased radiation capture (e.g. [25, 54]).

Besides their direct effect on plant growth, LEDs offer a lot of benefits compared to traditional lighting such as high-pressure sodium lamps including higher photon efficacy, longer operating times and energy savings while allowing spectral wavelength control [55–58]. The use of LEDs can straightforward reduce production costs of greenhouse growers. Although there are higher capital cost when installing a LED lighting system, the annual electricity costs are expected to recover the higher investment. LEDs can convert up to 50% of energy into usable light, whereas HPS lamps can only convert up to 30% resulting in significant energy savings when using LEDs [57]. Furthermore, LEDs do not emit as much heat as HPS lamps. This results in a comparable lower water consumption of crops produced under LEDs than HPS lamps [59] due to lower leaf temperature, and consequently transpiration rate [60]. However, it is worth noting, that the use of HPS lamps reduces costs during winter [61]. The use of efficient LEDs, however, requires optimized lighting strategies. This study can help, to minimize energy expenditure for supplemental lighting. As such, predictive models that incorporate sunlight predictions may further achieve a reduction in electrical energy of over 45% [62].

Narrowband LEDs are the preferred choice for supplemental lighting in greenhouse cultivation. However, although supplemental lighting enables compensation of adverse growing media effects it comes at a price, namely for energy.

### 4.3 Far-red light effects on plant growth, morphology and development depend on background PAR

The present study showed significant interactive effects between supplemental light dose and far-red light on leaf area and dry weight of Chinese cabbage (Fig 4D and 4E). These observations can mostly be traced back to and explained by spectral alternation within the light dose levels of our light gradient. Our light measurements revealed shifts in several light color proportions and ratios within the different PAR levels (Table 1), possibly caused by Raleigh’s scattering law [63], slight differences in reads at different light intensities or leftover noise produced during spectroradiometer reads. The ratios of red to far-red photons were rather constant within the light gradient and the proportion of far-red light was > 40% in all light dose treatments. However, at the same time we observed striking differences among absolute PAR levels (from 5 to 183 μmol m^-2^ s^-1^) and the ratio of far-red photons to PAR (from 1:1.5 to 1:2.0; Table 1). Hence, the found interactions on leaf number, dry weight and leaf area are likely not a consequence of changes in R/FR nor the proportion of far-red light within the light gradient but a result of changes in the FR/PAR. Far-red is known to enhance photochemical efficiency by balancing the excitation between photosystem I and photosystem II which synergistically increases photochemistry and photosynthesis [64]. It was demonstrated that quantum yield of photosystem II increases with the addition of far-red photons and that the effect was 8-fold greater under high PAR (750 μmol m^-2^ s^-1^) compared to low (50 μmol m^-2^ s^-1^) PAR under warm-white background light [65]. It was further indicated that the enhancement effect depends on the ratio of far-red to background PAR and that far-red photons hardly contribute to photosynthesis when shorter wavelengths are limited or non-existent [24]. These researches support our findings that far-red light is not effective when background PAR is scarce and becomes more effective under high background PAR (i.e. indicated by the interaction between light dose and far-red light; Fig 4B, 4D and 4E). We therefore deduce that under low PAR (<50 μmol m^-2^ s^-1^) PS I is not overexcited so that there is no synergistic effect of far-red light on photochemistry as far-red photons themselves are not photosynthetic active.

It should be noted that Legendre and van Iersel [66] explored the effects of far-red photons and PAR on plant morphology and physiology of lettuce just recently by establishing a perpendicular light gradient orthogonally to a far-red gradient. But they found no evidence for an interaction between far-red light and PAR. However, their PAR gradient did not cover low light doses (<100 μmol m^-2^ s^-1^). Therefore, their findings do not contradict our results. Instead, they complement each other shedding light on the potential interactive nature of far-red light and PAR.

## 5 Conclusions and prospects

From our study it can be concluded that

1. better external growing conditions (i.e., increased PAR) favor peat-based growing media as demonstrated by the interaction between growing medium and light dose;
2. supplemental lightning can compensate for adverse growing media effects influenced by plant species and season;
3. compensation by supplemental lighting is wavelength dependent (i.e., red light is the most efficient) making narrowband LEDs the preferred light source;
4. far-red light is not effective when background PAR is limited and becomes more effective under high background PAR

Besides our approach of using supplemental LED lighting to compensate for adverse growing media effects, potential solutions include the optimization of water and nutrient management when alternative growing media are used. Further research and testing are needed and will make larger substitutions of peat in growing media possible in the future.

## Supporting information

S1 Fig Schematic sketch of one block (replicate) of the RCBD with four replications. Two growing media, namely control (C) and substitute (S) were arranged within five supplemental LED light intensity levels (183, 111, 44, 15 and 5 μmol m^-2^ s^-1^). Light quality and growing media treatments were randomized in each replication.
(TIF)

S2 Fig Spectral distribution of light treatments. (TIF)

S3 Fig Sketch of the fluent supplemental light gradient. Average photosynthetic photon flux densities (PPFDs) and light quality proportions are given for each position within the light gradient.
(TIF)

S4 Fig Graphic illustration of how the compensation requirements of adverse growing media effects were estimated using basil and growing medium I as an example in three steps. First, ten plants were averaged to give one dry weight value per PAR level with 17 PAR levels in total (A). Then, dry weights were estimated by interpolating data assuming a linear relationship between dry weight and μmol PAR m^-2^ s^-1^ (dotted lines) (B). The points where the red lines intersect with the dotted line indicate the amount of PAR needed to reach the same dry weights as the control (C).
(TIF)

S5 Fig Effects of supplemental light dose and light color on light use efficiency (A), light interception (B) and leaf area per incident light (C, S4 Table) of Chinese cabbage grown under LEDs in a greenhouse. Different letters indicate significant differences among light quality treatments within light dose treatments. Levels of significance for light quality (LQ), light dose (LD) and their interaction (LQ x LD) are depicted (***, P<0.001). (***, P<0.001; **, P<0.01; *, P<0.05; ns, not significant). Error bars indicate SEM. Lines connecting observations are for visualization only.
(TIF)

S6 Fig Effects of supplemental growing media and light color on light use efficiency (A), light interception (B), leaf area per incident light (C), dry weight (D) and specific leaf area of Chinese cabbage grown under LEDs in a greenhouse. Different letters indicate significant differences among light quality treatments within growing media treatments.
(TIF)

S1 Table Nutrient levels and pH values of growing media. (DOCX)

S2 Table Data set of Experiments I and II. Average values of each treatment are shown.
(DOCX)

S3 Table ANOVA for growing media, light quality and light dose treatment effects and their interactions on plant growth, morphology and development of Chinese cabbage. (DOCX)

S4 Table Calculations of light interception, light use efficiency and leaf area. (DOCX)
