# Supplementary figures and images for: Compensation of adverse growing media effects on plant growth and morphology by supplemental LED lighting
Source: PLoS One. 2023 Sep 14;18(9):e0291601. doi: 10.1371/journal.pone.0291601 (PMC10501627; doi:10.1371/journal.pone.0291601)

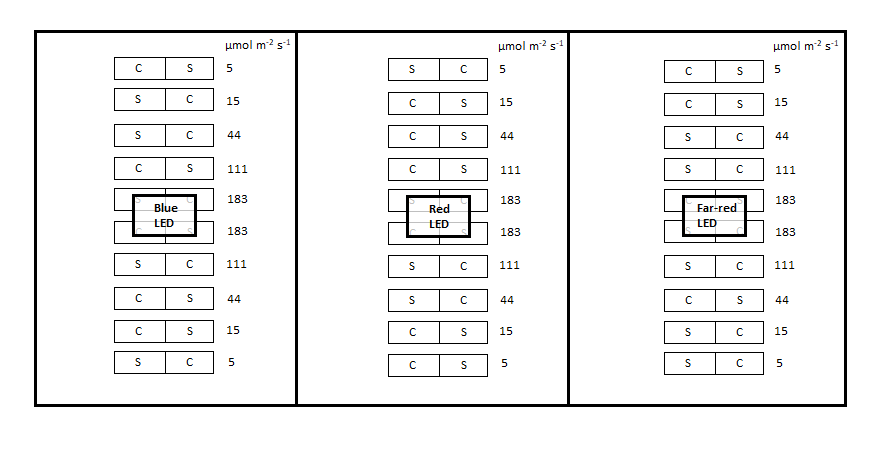

Supplement: S1 Fig — Two growing media, namely control (C) and substitute (S) were arranged within five supplemental LED light intensity levels (183, 111, 44, 15 and 5 μmol m-2 s-1). Light quality and growing media treatments were randomized in each replication. (TIF) [file pone.0291601.s001.tif]

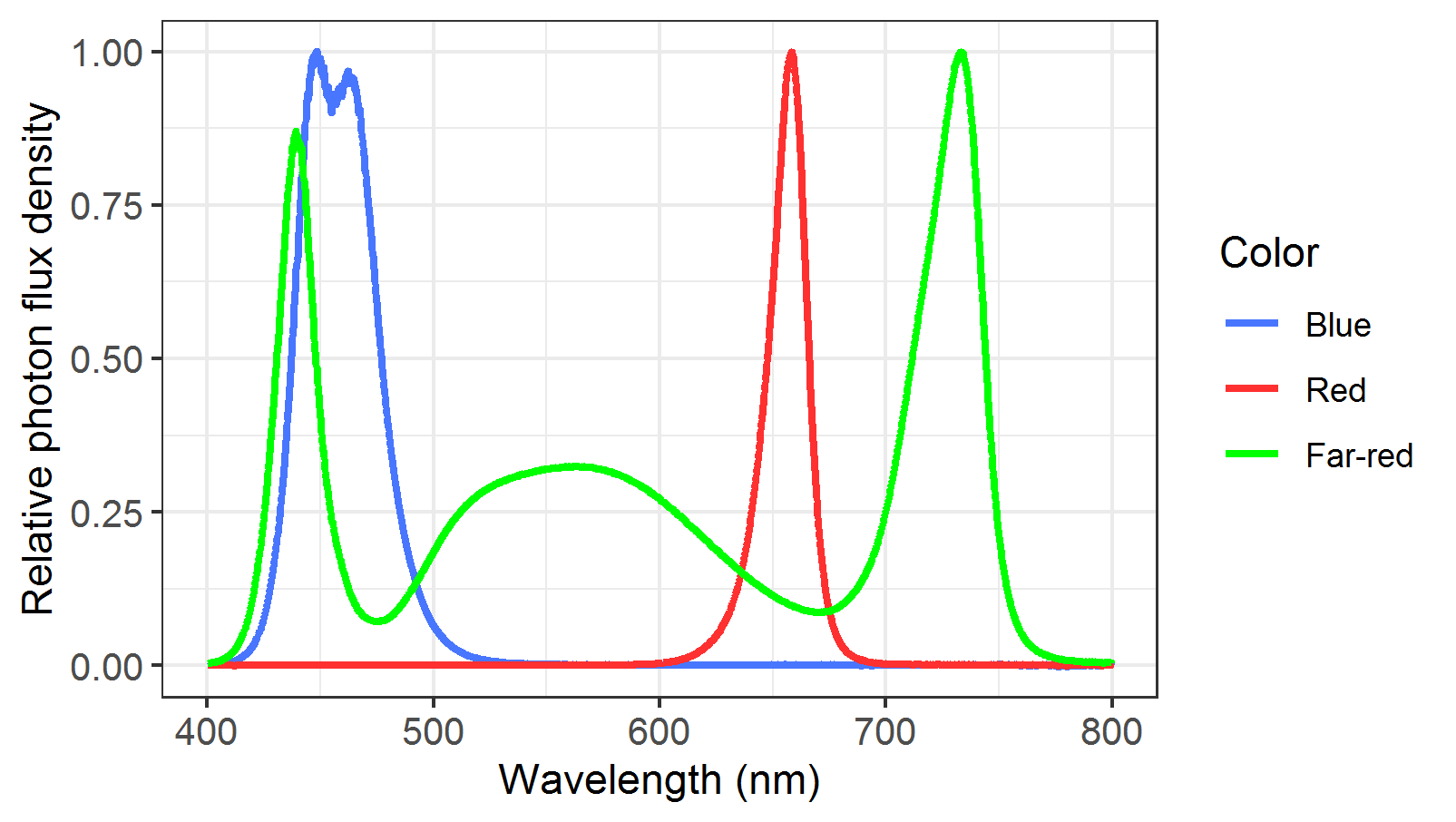

Supplement: S2 Fig — (TIF) [file pone.0291601.s002.tif]

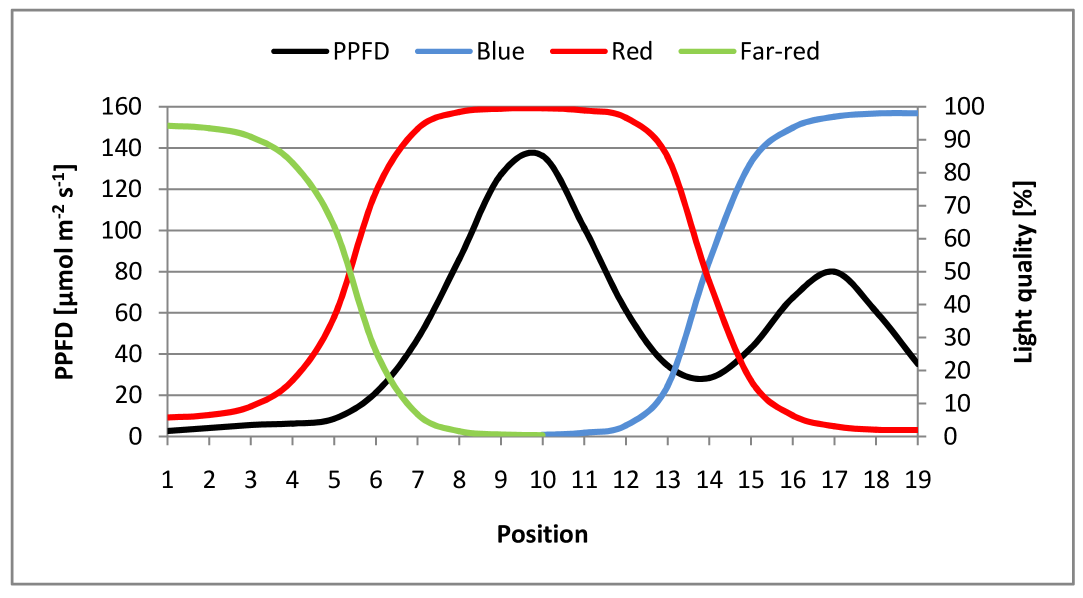

Supplement: S3 Fig — Average photosynthetic photon flux densities (PPFDs) and light quality proportions are given for each position within the light gradient. (TIF) [file pone.0291601.s003.tif]

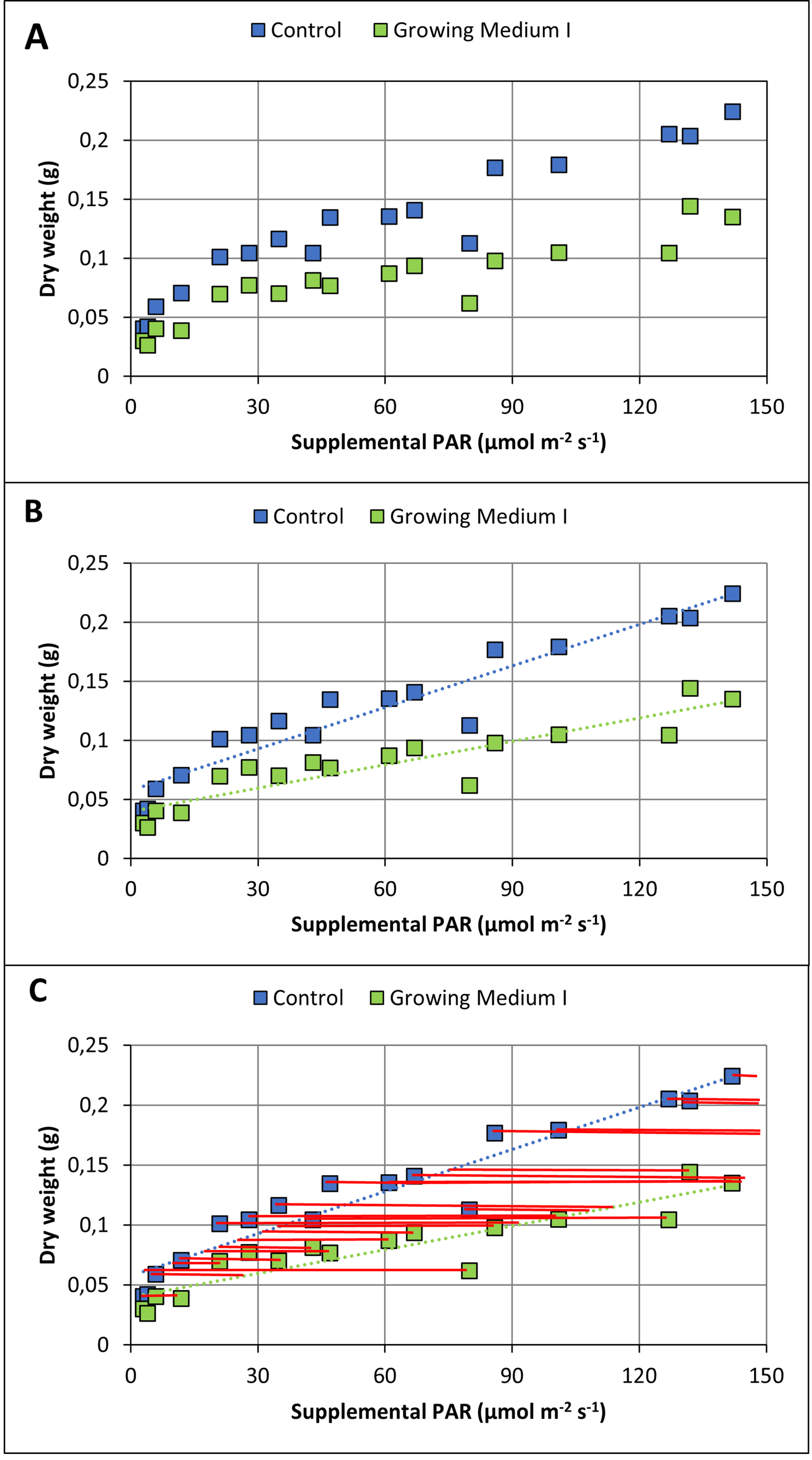

Supplement: S4 Fig — First, ten plants were averaged to give one dry weight value per PAR level with 17 PAR levels in total (A). Then, dry weights were estimated by interpolating data assuming a linear relationship between dry weight and μmol PAR m-2 s-1 (dotted lines) (B). The points where the red lines intersect with the dotted line indicate the amount of PAR needed to reach the same dry weights as the control (C). (TIF) [file pone.0291601.s004.tif]

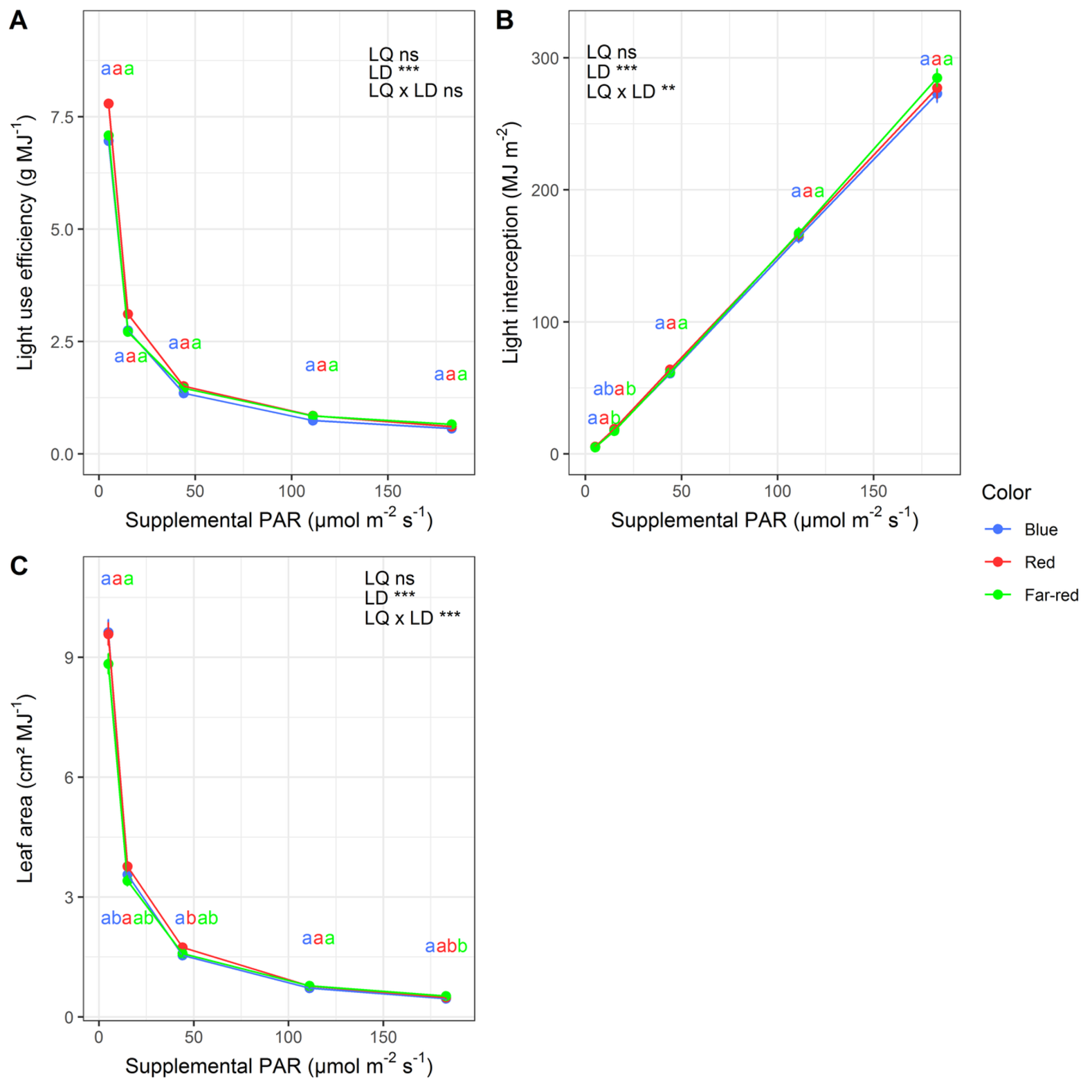

Supplement: S5 Fig — Different letters indicate significant differences among light quality treatments within light dose treatments. Levels of significance for light quality (LQ), light dose (LD) and their interaction (LQ x LD) are depicted (***, P<0.001). (***, P<0.001; **, P<0.01; *, P<0.05; ns, not significant). Error bars indicate SEM. Lines connecting observations are for visualization only. (TIF) [file pone.0291601.s005.tif]

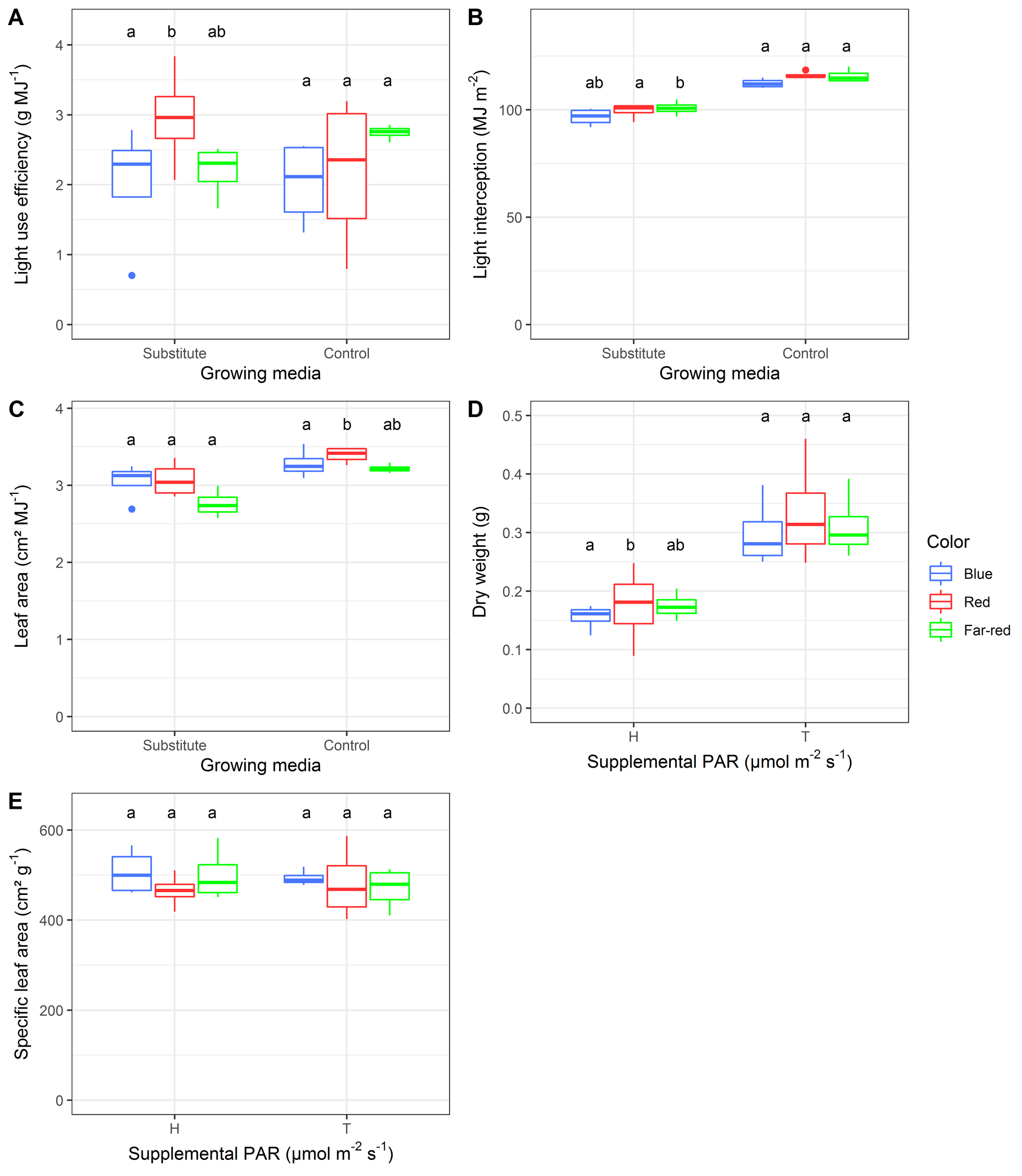

Supplement: S6 Fig — Different letters indicate significant differences among light quality treatments within growing media treatments. (TIF) [file pone.0291601.s006.tif]
