# Supplementary material for: Compensation of adverse growing media effects on plant growth and morphology by supplemental LED lighting
Source: PLoS One. 2023 Sep 14;18(9):e0291601. doi: 10.1371/journal.pone.0291601 (PMC10501627; doi:10.1371/journal.pone.0291601)
Supplement: S1 Table — (DOCX) [file pone.0291601.s007.docx]

**S1 Table. Nutrient levels and pH values of growing media**

| **Experiment I** | | | | | |
| --- | --- | --- | --- | --- | --- |
| **Growing media** | Nmin^x^  [mg L^-1^] | P_2_O_5_^y^  [mg L^-1^] | K_2_O^y^  [mg L^-1^] | Mg^y^  [mg L^-1^] | pH^z^ |
| Control | 207 | 141 | 230 | 333 | 5.6 |
| Substitute | 164 | 228 | 687 | 110 | 6.1 |
| **Experiment II** | | | | | |
| **Growing media** | Nmin^x^  [mg L^-1^] | P_2_O_5_^y^  [mg L^-1^] | K_2_O^y^  [mg L^-1^] | Mg^y^  [mg L^-1^] | pH^z^ |
| Control | 292 | 302 | 237 | 110 | 5.6 |
| Medium I | 333 | 384 | 322 | 164 | 5.8 |
| Medium II | 327 | 303 | 875 | 197 | 6.2 |

^x^ VDLUFA-Method, DIN 38406

^y^ CAT-Extraction after DIN EN 13651

^z^ VDLUFA-Method 5.2.1, 1991
