# Supplementary material for: Compensation of adverse growing media effects on plant growth and morphology by supplemental LED lighting
Source: PLoS One. 2023 Sep 14;18(9):e0291601. doi: 10.1371/journal.pone.0291601 (PMC10501627; doi:10.1371/journal.pone.0291601)
Supplement: S2 Table — Average values of each treatment are shown. (DOCX) [file pone.0291601.s008.docx]

**S2 Table. Data set of Experiments I and II. Average values of each treatment are shown.**

| **Experiment I** | | | | | | | | | | | |  |
| --- | --- | --- | --- | --- | --- | --- | --- | --- | --- | --- | --- | --- |
| **Light dose [µmol m^-2^ s^-1^]** | **Color** | | **Growing medium** | **Hypocotyl length [cm]** | | **Leaf number** | **Fresh weight [g]** | **Dry weight [g]** | | **SLA [cm² g^-1^]** | **Leaf area [cm²]** |  |
| 5 | Blue | | Substitute | 1.53 | | 4.6 | 1.81 | 0.089 | | 622.9 | 53.4 |  |
| 5 | Blue | | Control | 1.73 | | 4.8 | 1.79 | 0.081 | | 632.3 | 55.5 |  |
| 5 | Red | | Substitute | 1.2 | | 4.7 | 1.73 | 0.094 | | 545.9 | 51.5 |  |
| 5 | Red | | Control | 1.33 | | 5.2 | 1.76 | 0.097 | | 580.1 | 57.0 |  |
| 5 | Far-red | | Substitute | 2.44 | | 4.0 | 1.51 | 0.073 | | 535.6 | 39.5 |  |
| 5 | Far-red | | Control | 2.64 | | 4.7 | 1.79 | 0.086 | | 591.7 | 51.2 |  |
| 15 | Blue | | Substitute | 1.65 | | 4.82 | 2.24 | 0.10 | | 563.1 | 62.6 |  |
| 15 | Blue | | Control | 1.65 | | 5.21 | 2.56 | 0.11 | | 638.3 | 69.9 |  |
| 15 | Red | | Substitute | 0.85 | | 5.06 | 2.50 | 0.12 | | 508.5 | 68.0 |  |
| 15 | Red | | Control | 1.14 | | 5.80 | 2.76 | 0.13 | | 618.62 | 77.52 |  |
| 15 | Far-red | | Substitute | 2.66 | | 4.50 | 2.25 | 0.09 | | 515.8 | 51.7 |  |
| 15 | Far-red | | Control | 3.08 | | 5.19 | 2.84 | 0.11 | | 621.0 | 70.3 |  |
| 44 | Blue | | Substitute | 1.47 | | 5.02 | 3.12 | 0.13 | | 525.4 | 77.9 |  |
| 44 | Blue | | Control | 1.43 | | 6.14 | 5.22 | 0.23 | | 528.3 | 114.7 |  |
| 44 | Red | | Substitute | 0.40 | | 5.91 | 3.60 | 0.19 | | 457.9 | 91.9 |  |
| 44 | Red | | Control | 0.50 | | 7.06 | 5.44 | 0.23 | | 555.7 | 136.0 |  |
| 44 | Far-red | | Substitute | 2.39 | | 5.10 | 3.59 | 0.17 | | 526.3 | 83.4 |  |
| 44 | Far-red | | Control | 2.94 | | 5.97 | 5.70 | 0.23 | | 473.9 | 118.3 |  |
| 111 | Blue | | Substitute | 1.07 | | 5.46 | 3.90 | 0.18 | | 456.8 | 92.7 |  |
| 111 | Blue | | Control | 1.21 | | 6.68 | 7.07 | 0.37 | | 428.3 | 149.6 |  |
| 111 | Red | | Substitute | 0.23 | | 6.37 | 4.11 | 0.25 | | 429.3 | 97.7 |  |
| 111 | Red | | Control | 0.22 | | 7.85 | 7.02 | 0.40 | | 467.0 | 168.6 |  |
| 111 | Far-red | | Substitute | 1.80 | | 5.56 | 4.27 | 0.21 | | 468.3 | 97.5 |  |
| 111 | Far-red | | Control | 2.19 | | 6.71 | 8.28 | 0.44 | | 400.3 | 169.6 |  |
| 183 | Blue | | Substitute | 0.81 | | 5.80 | 4.07 | 0.23 | | 365.1 | 92.7 |  |
| 183 | Blue | | Control | 0.98 | | 6.89 | 8.00 | 0.46 | | 377.1 | 164.9 |  |
| 183 | Red | | Substitute | 0.21 | | 6.72 | 4.5 | 0.30 | | 368.1 | 102.6 |  |
| 183 | Red | | Control | 0.18 | | 8.08 | 7.6 | 0.469 | | 403.2 | 179.1 |  |
| 183 | Far-red | | Substitute | 1.37 | | 6.16 | 5.36 | 0.28 | | 437.5 | 116.8 |  |
| 183 | Far-red | | Control | 1.58 | | 7.25 | 9.14 | 0.56 | | 352.7 | 190.1 |  |
|  | | | | | | | | | | | |  |
| **Experiment II** | | | | | | | | | | | |  |
| **PAR level [µmol m^-2^ s^-1^]** | | **Control** | | | **Growing medium I** | | | | **Growing medium II** | | | |
|  | | **Chinese cabbage - Spring**  ) | | | | | | | | | | |
| 2.7 | | 0.0707 | | | 0.0714 | | | | 0.0682 | | | |
| 4.1 | | 0.0766 | | | 0.0768 | | | | 0.0706 | | | |
| 5.6 | | 0.0807 | | | 0.0806 | | | | 0.0685 | | | |
| 6.3 | | 0.0821 | | | 0.0801 | | | | 0.0718 | | | |
| 8.7 | | 0.0825 | | | 0.0836 | | | | 0.0787 | | | |
| 21.4 | | 0.0977 | | | 0.1046 | | | | 0.0928 | | | |
| 28.4 | | 0.1061 | | | 0.1048 | | | | 0.1035 | | | |
| 34.5 | | 0.1099 | | | 0.1119 | | | | 0.1094 | | | |
| 35.2 | | 0.1059 | | | 0.1142 | | | | 0.1054 | | | |
| 42.9 | | 0.1147 | | | 0.1211 | | | | 0.1128 | | | |
| 47.3 | | 0.1266 | | | 0.1254 | | | | 0.1195 | | | |
| 60.7 | | 0.1258 | | | 0.1157 | | | | 0.1102 | | | |
| 61.1 | | 0.1228 | | | 0.1300 | | | | 0.1246 | | | |
| 67.3 | | 0.1349 | | | 0.1238 | | | | 0.1208 | | | |
| 79.9 | | 0.1166 | | | 0.1267 | | | | 0.1332 | | | |
| 86.0 | | 0.1368 | | | 0.1295 | | | | 0.1264 | | | |
| 101.2 | | 0.1221 | | | 0.1302 | | | | 0.1306 | | | |
| 127.2 | | 0.1679 | | | 0.1519 | | | | 0.1675 | | | |
| 136.2 | | 0.1435 | | | 0.1390 | | | | 0.1546 | | | |
|  | | **Chinese cabbage - Summer** | | | | | | | | | | |
| 2.7 | | 0.1202 | | | 0.0730 | | | | 0.1035 | | | |
| 4.1 | | 0.1266 | | | 0.0971 | | | | 0.0903 | | | |
| 5.6 | | 0.1432 | | | 0.1038 | | | | 0.0989 | | | |
| 6.3 | | 0.1459 | | | 0.1051 | | | | 0.0994 | | | |
| 8.7 | | 0.1353 | | | 0.1064 | | | | 0.1078 | | | |
| 21.4 | | 0.1498 | | | 0.1064 | | | | 0.1274 | | | |
| 28.4 | | 0.1549 | | | 0.0811 | | | | 0.1313 | | | |
| 34.5 | | 0.1504 | | | 0.0923 | | | | 0.1367 | | | |
| 35.2 | | 0.1611 | | | 0.1172 | | | | 0.1314 | | | |
| 42.9 | | 0.1604 | | | 0.1143 | | | | 0.1594 | | | |
| 47.3 | | 0.1638 | | | 0.0920 | | | | 0.1377 | | | |
| 60.7 | | 0.1644 | | | 0.1361 | | | | 0.1444 | | | |
| 61.1 | | 0.1632 | | | 0.0947 | | | | 0.1495 | | | |
| 67.3 | | 0.1688 | | | 0.1128 | | | | 0.1780 | | | |
| 79.9 | | 0.1772 | | | 0.0892 | | | | 0.1461 | | | |
| 86.0 | | 0.1821 | | | 0.1286 | | | | 0.1621 | | | |
| 101.2 | | 0.1750 | | | 0.1156 | | | | 0.1843 | | | |
| 127.2 | | 0.2114 | | | 0.0784 | | | | 0.1855 | | | |
| 136.2 | | 0.2084 | | | 0.0950 | | | | 0.1816 | | | |
|  | | **Basil - Summer** | | | | | | | | | | |
| 3 | | 0.1836 | | | 0.1063 | | | | 0.11877 | | | |
| 4 | | 0.1918 | | | 0.1178 | | | | 0.154 | | | |
| 6 | | 0.2125 | | | 0.1172 | | | | 0.1158 | | | |
| 12 | | 0.2223 | | | 0.1003 | | | | 0.1288 | | | |
| 21 | | 0.2478 | | | 0.1822 | | | | 0.1384 | | | |
| 28 | | 0.2351 | | | 0.1446 | | | | 0.1611 | | | |
| 35 | | 0.2502 | | | 0.1393 | | | | 0.181 | | | |
| 43 | | 0.2556 | | | 0.1282 | | | | 0.1683 | | | |
| 47 | | 0.2787 | | | 0.182 | | | | 0.1544 | | | |
| 61 | | 0.2687 | | | 0.1717 | | | | 0.1767 | | | |
| 67 | | 0.2868 | | | 0.1394 | | | | 0.1829 | | | |
| 80 | | 0.2963 | | | 0.1616 | | | | 0.1613 | | | |
| 86 | | 0.2782 | | | 0.2159 | | | | 0.1846 | | | |
| 101 | | 0.3168 | | | 0.1649 | | | | 0.1981 | | | |
| 127 | | 0.3776 | | | 0.1717 | | | | 0.2113 | | | |
| 132 | | 0.3447 | | | 0.2356 | | | | 0.2083 | | | |
| 142 | | 0.366 | | | 0.1944 | | | | 0.2325 | | | |
|  | | **Basil - Autumn** | | | | | | | | | | |
| 3 | | 0.0404 | | | 0.03 | | | | 0.0341 | | | |
| 4 | | 0.0418 | | | 0.0261 | | | | 0.0358 | | | |
| 6 | | 0.0589 | | | 0.0404 | | | | 0.0475 | | | |
| 12 | | 0.0702 | | | 0.0387 | | | | 0.0612 | | | |
| 21 | | 0.1009 | | | 0.0694 | | | | 0.091 | | | |
| 28 | | 0.1042 | | | 0.077 | | | | 0.0894 | | | |
| 35 | | 0.1162 | | | 0.0699 | | | | 0.0954 | | | |
| 43 | | 0.1041 | | | 0.081 | | | | 0.0847 | | | |
| 47 | | 0.1345 | | | 0.0765 | | | | 0.1053 | | | |
| 61 | | 0.135 | | | 0.0869 | | | | 0.1103 | | | |
| 67 | | 0.1407 | | | 0.0935 | | | | 0.126 | | | |
| 80 | | 0.1125 | | | 0.0616 | | | | 0.1138 | | | |
| 86 | | 0.1765 | | | 0.0977 | | | | 0.1374 | | | |
| 101 | | 0.1791 | | | 0.1048 | | | | 0.1377 | | | |
| 127 | | 0.2051 | | | 0.1044 | | | | 0.1698 | | | |
| 132 | | 0.2032 | | | 0.1438 | | | | 0.1751 | | | |
| 142 | | 0.2239 | | | 0.1347 | | | | 0.1773 | | | |
