# Supplementary material for: Compensation of adverse growing media effects on plant growth and morphology by supplemental LED lighting
Source: PLoS One. 2023 Sep 14;18(9):e0291601. doi: 10.1371/journal.pone.0291601 (PMC10501627; doi:10.1371/journal.pone.0291601)
Supplement: S3 Table — (DOCX) [file pone.0291601.s009.docx]

**S3 Table. ANOVA for growing media, light quality and light dose treatment effects and their interactions on plant growth, morphology and development of Chinese cabbage.**

| **Source** | **Trait** | **GM** | **LQ** | **LD** | **GM x LD** | **LQxLD** |
| --- | --- | --- | --- | --- | --- | --- |
| **dF**  **F value**  **Significance** | Hypocotyl length | 1  58  *** | 2  470.9  *** | 4  128.6  *** | 4  0.83  NS | 8  11.825  *** |
| **dF**  **F value**  **Significance** | Leaf number | 1  301.4  *** | 2  4.501  . | 4  230.7  *** | 4  6.587  *** | 8  3.524  ** |
| **dF**  **F value**  **Significance** | Fresh weight | 1  388.4  *** | 2  1.925  NS | 4  382.3  *** | 4  26.964  *** | 8  1.786  NS |
| **dF**  **F value**  **Significance** | Dry weight | 1  242.1  *** | 2  2.794  NS | 4  519.4  *** | 4  25.618  *** | 8  2.632  * |
| **dF**  **F value**  **Significance** | Leaf area | 1  495.2  *** | 2  3.018  NS | 4  348.3  *** | 4  22.982  *** | 8  4.388  *** |
| **dF**  **F value**  **Significance** | Specific leaf area | 1  0.029  NS | 2  0.52  NS | 4  62  *** | 4  2.745  * | 8  1.245  NS |

GM: Growing media, LQ: Light quality, LD: Light dose, GMxLD: Growing Mediax Light dose interaction, LQxLD: Light quality x Light dose interaction; Significance: '***' 0.001; '**' 0.01; '*' 0.05; '.' 0.1; 'NS' not significant
