# Supplementary material for: Compensation of adverse growing media effects on plant growth and morphology by supplemental LED lighting
Source: PLoS One. 2023 Sep 14;18(9):e0291601. doi: 10.1371/journal.pone.0291601 (PMC10501627; doi:10.1371/journal.pone.0291601)
Supplement: S4 Table — (DOCX) [file pone.0291601.s010.docx]

**S4 Table. Calculations of light interception, light use efficiency and leaf area.**

| **Parameter** | **Description** |
| --- | --- |
| Light interception (MJ m^-2^) | First, the amount of daily intercepted light (Q_daily_, MJ m^-2^ d^-1^) by a plant was calculated following Beer-Lambert law  $Q_{daily}=I*(1-e^{-k*LAI})$  where *I* is the amount of daily light above the plant (sum of natural and supplemental PAR, MJ m^-2^ d^-1^), *LAI* is the leaf area index (m^2^ leaf area per m^-2^ ground area) and *k* is the light extinction coefficient (assumed as 0.8).  Since leaf areas could only be measured at final harvest, leaf areas between the start of the supplemental light treatment and final harvest were estimated based on two data points: the start of the supplemental light treatment and final harvest. The two data points were log-transformed, and a linear regression was fitted to the data to estimate leaf areas for each day beginning from the start of the supplemental light treatment. Afterwards, leaf areas were back transformed to follow an exponential function.  The total amount of light intercepted (Q_total_, MJ m^-2^) of a plant was subsequently calculated by summing up Q_daily_ until final harvest.  Photosynthetic quantum flux (µmol m^-2^ s^-1^) was integrated over the light period and using a conversion factor of 0.219 to give the daily light energy flux (MJ PAR m^-2^ s^-1^). |
| Light use efficiency (g MJ^-1^) | Light use efficiency (g MJ^-1^) was calculated by dividing a plants dry weight at harvest (g m^-2^) by the total amount absorbed light (Q_total_, MJ m^-2^). |
| Leaf area  (cm^2^ MJ^-1^) | Leaf area per absorbed light (cm^2^ MJ^-1^) was calculated by dividing a a plants leaf area at harvest by the total amount absorbed light (Q_total_, MJ m^-2^). |
